# Reveromycin A Administration Prevents Alveolar Bone Loss in Osteoprotegerin Knockout Mice with Periodontal Disease

**DOI:** 10.1038/srep16510

**Published:** 2015-11-12

**Authors:** Manami Mizuno, Ken Miyazawa, Masako Tabuchi, Miyuki Tanaka, Mamoru Yoshizako, Chisato Minamoto, Yasuyoshi Torii, Yusuke Tamaoka, Makoto Kawatani, Hiroyuki Osada, Hatsuhiko Maeda, Shigemi Goto

**Affiliations:** 1 Department of Orthodontics, School of Dentistry, Aichi Gakuin University, Nagoya, Aichi 464-8651, Japan; 2 Chemical Biology Research Group, RIKEN CSRS, Wako, Saitama 351-0198, Japan; 3 Department of Oral Pathology, School of Dentistry, Aichi Gakuin University, Nagoya, Aichi 464-8650, Japan

## Abstract

Chronic periodontal disease is characterized by alveolar bone loss and inflammatory changes. Reveromycin A (RMA) was recently developed and is a unique agent for inhibiting osteoclast activity. This study analysed the effects of RMA in an experimental mouse model of periodontitis involving osteoprotegerin (OPG)-knockout mice, specifically, whether it could control osteoclasts and reduce inflammation in periodontal tissue. We examined wild-type (WT) and OPG knockout mice (OPG KO) ligated with wire around contact points on the left first and second molars. RMA was administered twice a day to half of the mice. Using micro-computed tomography, we measured the volume of alveolar bone loss between the first and second molars, and also performed histological analysis. The OPG KO RMA+ group had significantly decreased osteoclast counts, alveolar bone loss, attachment loss, and inflammatory cytokine expression 8 weeks after ligation. Thus, RMA may reduce alveolar bone loss and inflamed periodontal tissues in patients with periodontitis.

In bone remodelling of periodontal tissue, osteoclasts resorb bone and osteoblasts form new bone. Substantial bone resorption is observed in patients with periodontitis, which is caused by a build-up of dental plaque and release of inflammatory mediators, due to weakened periodontal tissue and an imbalance in bone metabolism^1^,^2^,^3^. Systematic diseases such as osteoporosis are also related to bone resorption, and in Japanese women, low bone mineral density leads to progression of periodontal disease and tooth loss^2^. A previous study has demonstrated that orthodontic therapy performed without treating periodontitis resulted in substantial bone resorption^4^. It is important to keep the periodontal tissue free of periodontitis and below the clinical pocket depth of 5–6 mm^5^.

The processes of osteoclast differentiation, maturation, and functioning are mediated by receptor activator superfamily of NF-κB ligand (RANKL), which is expressed on the cell membranes of both osteoblasts and bone marrow stromal cells^6^. Osteoclasts and their precursor cells express receptor activator of NF-κB (RANK), which interacts with RANKL via intercellular contact, inducing the differentiation of precursor into osteoclasts^7^,^8^,^9^. In addition to RANKL, osteoblasts produce osteoprotegerin (OPG), a member of the tumour necrosis factor superfamily. OPG acts as a decoy receptor, binding to RANKL and inhibiting the RANK–RANKL interaction. OPG also acts to strongly inhibit the RANKL–RANK interaction, suppressing osteoclast differentiation and functional expression. *In vivo*, OPG-overexpressing mice have reduced bone resorption and develop severe osteopetrosis^10^. Conversely, bone in osteoprotegerin knockout (OPG KO) mice appears normal at birth, but osteoclast activity is promoted as the mice grow. Adult OPG KO mice have lower bone mineral density, characterized by severe trabecular and cortical bone porosity and a high incidence of fracture^11^,^12^. These OPG KO mice are a useful model for osteoporosis and periodontology^13^.

At present, periodontal disease is treated using minocycline, an antibiotic. This is effective in reducing bacterial counts^14^,^15^, but minocycline does not affect osteoclasts or inhibit bone resorption directly. Recently, the drug reveromycin A (RMA), an acidic compound produced by *Streptomyces reveromyceticus*, was developed to selectively suppress osteoclast activity. This compound is selectively absorbed by osteoclasts that are actively secreting acid and dissolving bone, but it is not absorbed by regular cells. RMA can affect osteoclasts directly to inhibit bone resorption by selectively controlling apoptosis of activated osteoclasts^16^,^17^,^18^. In this study, we aimed to identify the effects of RMA in periodontal tissue, using an experimental model of periodontitis caused by food impaction, established using ligature wire tied around the contact point between the left maxillary first molar (M1) and second molar (M2)^19^. We examined alveolar bone remodeling in OPG KO mice and wild-type (WT) mice and hypothesized that the administration of RMA would inhibit alveolar bone resorption controlled by osteoclasts.

## Results

### Micro CT findings and comparison of the ratio of remaining alveolar bone

**Figure 1:**
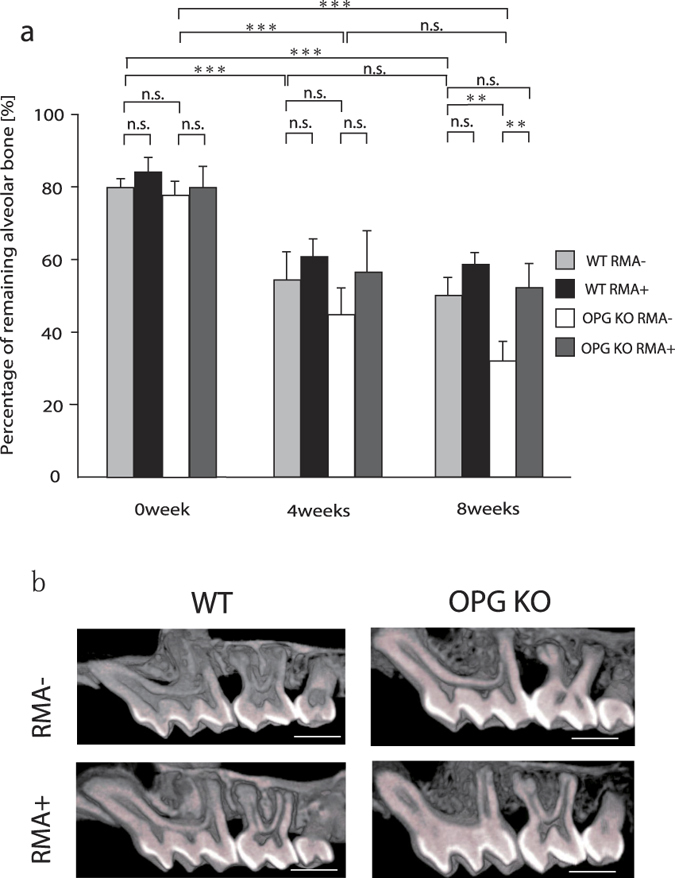
Microfocus x-ray CT findings between M1and M2 in mice. (**a**) Remaining alveolar bone (%), n.s., not significant; **p < 0.01; ***p < 0.001, (**b**) Representative micro-computed tomography images of the alveolar bone of test and control maxillae at 0 days, 4 weeks, and 8 weeks (buccal view), Scale Bar = 1000 μm Abbreviations: WT, wild-type mice; OPG KO, OPG knockout mice; RMA+, given reveromycin A; RMA–, not given reveromycin A.

Both WT mice given (RMA+) or not given RMA (RMA–) showed alveolar bone loss over time from 0 week to 4 weeks and 8 weeks after ligature wire was placed (p > 0.001). The same results were seen in OPG KO RMA+ and RMA– mice (p > 0.001) (Fig. 1a). There was no significant difference in the proportion of remaining alveolar bone at 4 weeks after ligature between all groups. However, there was a significantly higher proportion of remaining alveolar bone at 8 weeks after ligature in OPG KO RMA+ compared with OPG KO RMA– mice (p > 0.01). A higher proportion of remaining alveolar bone tended to be seen in both WT RMA+ and WT RMA– mice, but the two groups did not significantly differ at 4 weeks and 8 weeks. There was no significant difference between the OPG KO RMA+ and WT RMA– groups at 8 weeks after ligature (p < 0.05) (Fig. 1a). Figure 1b shows the representative microfocus X-ray CT images of the alveolar bone in each group.

### Pathological findings and attachment loss

**Figure 2:**
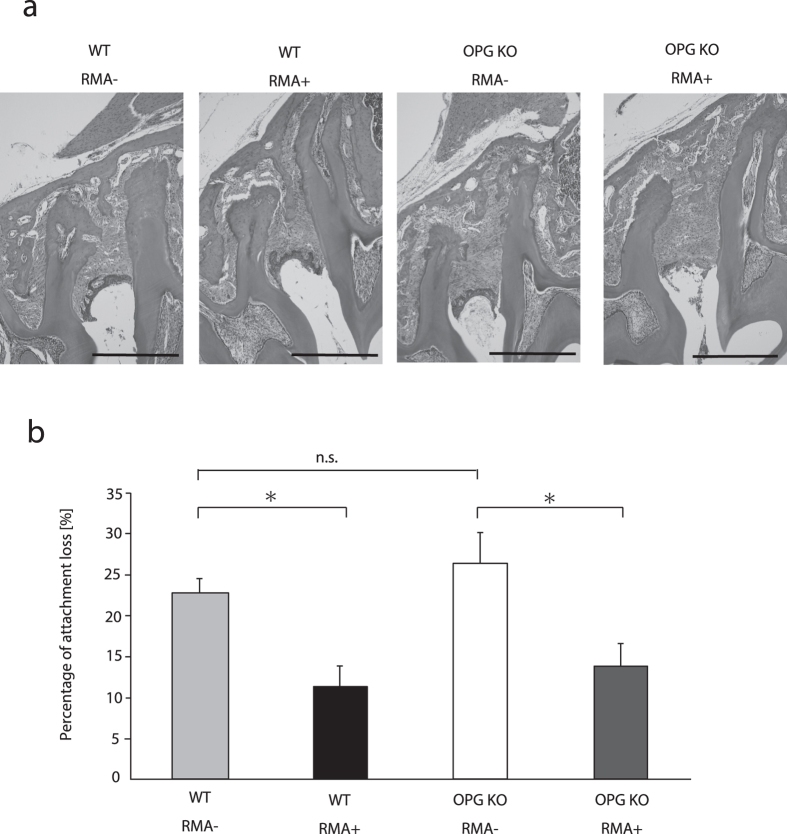
Histological and histomorphometric analyses between M1 and M2 in mice. (**a**) Representative hematoxylin-eosin stained periodontium between the first and second molars at 8 weeks after ligature wire was placed. Bar = 500 μm, original magnification × 100. (**b**) The percentage of attachment loss from cementonamel junction to the apex of the root 8 weeks after placement of ligature wire (%) n.s.: not significant, *p < 0.05 Abbreviations: WT, wild-type mice; OPG KO, OPG knockout mice; RMA+, given reveromycin A; RMA–, not given reveromycin A.

Histologic findings in the periodontal tissue indicated that the attachment loss in the region from the cementoenamel junction to the root apex was significantly lower in the OPG KO RMA+ group than in the OPG KO RMA– group at 8 weeks after placement of ligature wire (p < 0.05) (Fig. 2). In the WT groups after 8 weeks, RMA+ groups had significantly lower attachment loss than RMA– groups (p < 0.05) (Fig. 2).

### Osteoclast count

**Figure 3:**
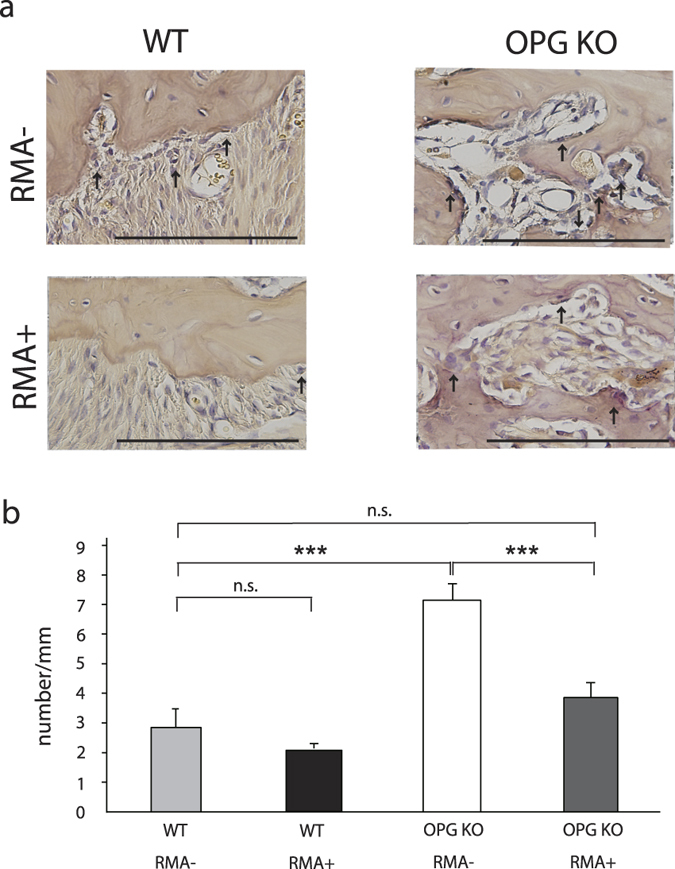
Expression of osteoclasts between M1 and M2 in mice. (**a**) Osteoclasts (arrows) along the bone surface between the first and second molars at 8 weeks after ligature wire was placed (Bar = 200 μm; original magnification, 400 × ; ↑:osteoclasts), (**b**) Osteoclast count (number/mm). n.s., not significant; ***p < 0.001, Abbreviations: WT, wild-type mice; OPG KO, OPG knockout mice; RMA+, given reveromycin A; RMA–, not given reveromycin A.

Osteoclast counts of the WT RMA+ group at 8 weeks were somewhat, but not significantly, lower than those in the WT RMA– group (Fig. 3). RMA administration led to a significant decrease in osteoclast count in the OPG KO groups; the OPG KO RMA– group had high osteoclast counts (p < 0.001). Osteoclast counts did not significantly differ between the OPG KO RMA+ and WT RMA– groups (Fig. 3).

### Inflammatory cytokine expression

**Figure 4:**
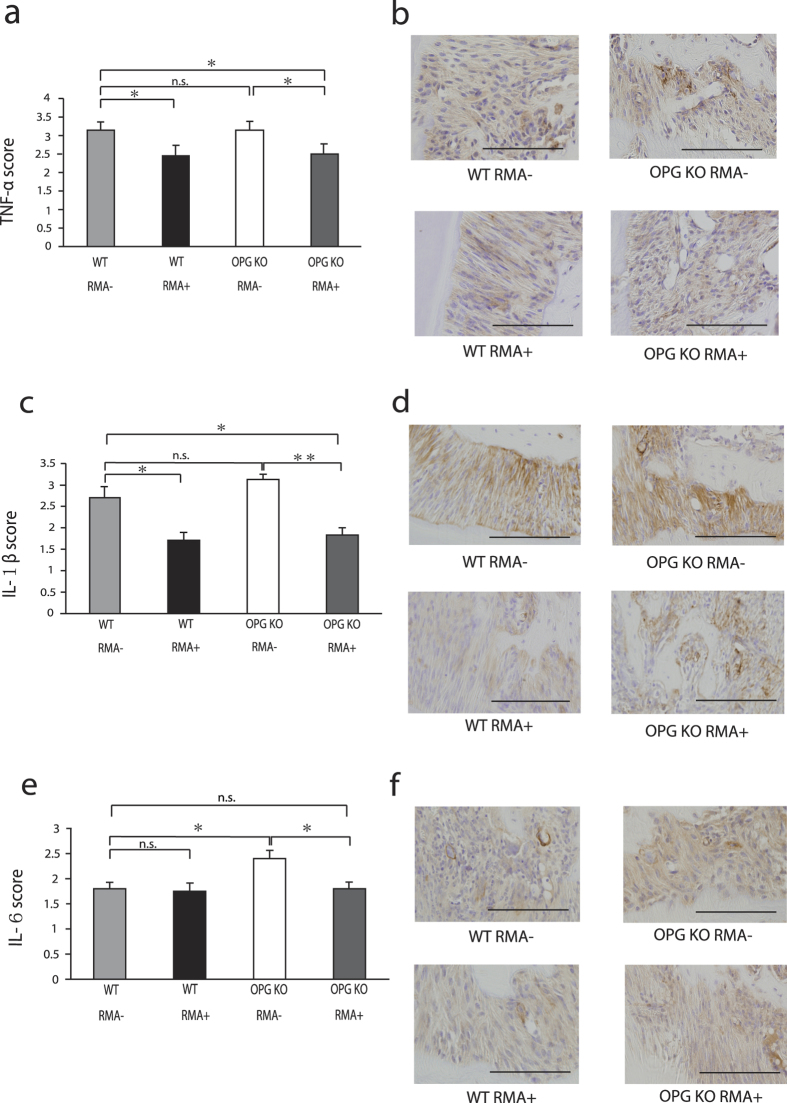
Immunohistochemical analyses between M1 and M2 in mice. (**a**) Immunohistochemical scores of TNF-α, (**b**) immunostaining of TNF-α, (**c**) immunohistochemical scores of IL-1β, (**d**) Immunostaining of IL-1β, (**e**) immunohistochemical scores of IL-6, and (**f**) immunostaining of IL-6 at 8 weeks after ligature wire was placed. n.s., not significant; *p < 0.05; **p < 0.01, Bar = 100 μm, original magnification × 400. Abbreviations: WT, wild-type mice; OPG KO, OPG knockout mice; RMA+, given reveromycin A; RMA–, not given reveromycin A.

Immunostaining showed expression of inflammatory cytokines and cells in the periodontal ligament. The TNF-α and IL-1β scores of the WT RMA+ group were significantly lower than those in the WT RMA– group at 8 weeks after placement of ligature wire (p < 0.05) (Fig. 4). In the OPG KO groups, RMA administration significantly lowered the scores for TNF-α (p < 0.05), IL-1β (p < 0.01), and IL-6 (p < 0.05) (Fig. 4).

## Discussion

Periodontitis is currently treated by antibiotics only such as minocycline hydrochloride^14^,^15^. These medications do not act on osteoclasts directly to prevent or suppress bone resorption. Bisphosphonates, on the other hand, are synthetic analogues of pyrophosphate that act directly on osteoclasts to inhibit bone resorption^20^,^21^,^22^ and can thus be used to treat osteoporosis. However, recent research on bisphosphonate-related osteonecrosis of the jaw with surgical dental treatment has shown that bisphosphonates have adverse effects that make them unsuitable for long-term therapy^23^,^24^,^25^,^26^. Therefore, we focused on RMA in the present study, specifically, an acidic substance produced by *Streptomyces reveromyceticus*. RMA does not affect inexperienced progenitor cells and inactivated osteoclasts, which have no bone-resorbing ability, but instead selectively affect osteoclasts that are actively secreting acid and dissolving bone^17^. Furthermore, being itself an acidic compound with three carboxylic acid moieties, RMA is selectively taken up by active osteoclasts, which secrete acids to dissolve bones, within acidic environments^16^,^17^,^18^.

Yabumoto *et al.* performed experimental tooth movement according to the Waldo method to investigate the role of RMA, and revealed that RMA and bisphosphonates had similar effects^27^. In a different study using a mice experimental model of tooth movement and super-elastic orthodontic coil springs, Tanaka *et al.* performed long-term observation for a period longer than that in the Waldo method, and reported the normalization of tooth movement by RMA, similarly to that by biphosphonates^28^.

The present study revealed a significant increase in osteoclast counts and alveolar bone loss in OPG KO RMA– mice. This suggests that in OPG KO mice, the net-like structure of cancellous bone is congenitally weak and osteoclasts are highly activated, creating an ideal environment for alveolar bone resorption. Here, already active osteoclasts in OPG KO mice were further activated by the ligature wire placed at the interproximal contact point, unbalancing bone metabolism. This indicates that unless appropriate systemic and local treatments are provided, pathological alveolar bone resorption and dental attachment loss will occur due to enhanced osteoclast activity in patients with primary type 1 osteoporosis and in those with extremely weak periodontal tissues.

In contrast, a significant reduction in osteoclast counts and bone resorption in the alveolar septum were observed in OPG KO RMA+ mice 8 weeks after placement of ligature wire. Furthermore, the administration of RMA in WT mice was able to reduce, albeit not significantly, osteoclast counts and dental attachment loss in the area between the cementoenamel junction and root apex, suggesting that RMA inhibits osteoclast activation and alveolar bone resorption.

Because bone resorption in periodontitis is induced by inflammatory cytokines IL-1, IL-6, and TNF-α^29^, the suppression of these inflammatory cytokines may be the key to successful treatment of periodontitis. In this study, the administration of RMA significantly reduced the scores of IL-1β, IL-6, and TNF-α in the OPG KO RMA+ mice compared with OPG KO RMA–– mice, suggesting that RMA not only suppresses the activity of osteoclasts directly, but also augments this suppression by inhibiting inflammatory cytokines.

Despite their similar efficacies, RMA and bisphosphonates have different mechanisms of action. Unlike bisphosphonates, RMA does not remain in bone for a long period of time or cause adverse effects or osteonecrosis of the jaw following tooth extraction, which enables it to be administered over the long term. In addition, oral RMA may be administered at high doses without any concern for adverse systemic reactions because the drug is inactivated and hydrolysed by stomach acid^17^. As in the OPG KO mice in this study, RMA may prevent the weakening of periodontal tissues and deter the progression of periodontitis in patients with primary type 1 osteoporosis and those with extremely weak periodontal tissues through the suppression and prevention of pathological alveolar bone resorption. Although further investigation is necessary, this may reveal a new role for RMA as a local drug for periodontitis.

## Methods

### Animals

A total of 48 eight-week-old male OPG KO (n = 4) and WT (C57BL/6J, n = 4) mice were used in this study (CLEA Japan, Osaka, Japan) and housed in the animal experimentation laboratory of the School of Dentistry, Aichi-Gakuin University, Japan. Room temperature and humidity were maintained at 22 ± 2 °C and 50 ± 10%, respectively, and a 12-hour light/dark cycle was established. Mice had free access to solid food (CE-2; CLEA Japan, Tokyo, Japan) and tap water. Animal care and experimental procedures were in accordance with the Guidelines for Animal Experiments of the School of Dentistry, Aichi-Gakuin University and all experiments were approved by the Animal Experimental Committee at the Dentistry of Aichi Gakuin University (AGUD 103).

**Figure 5:**
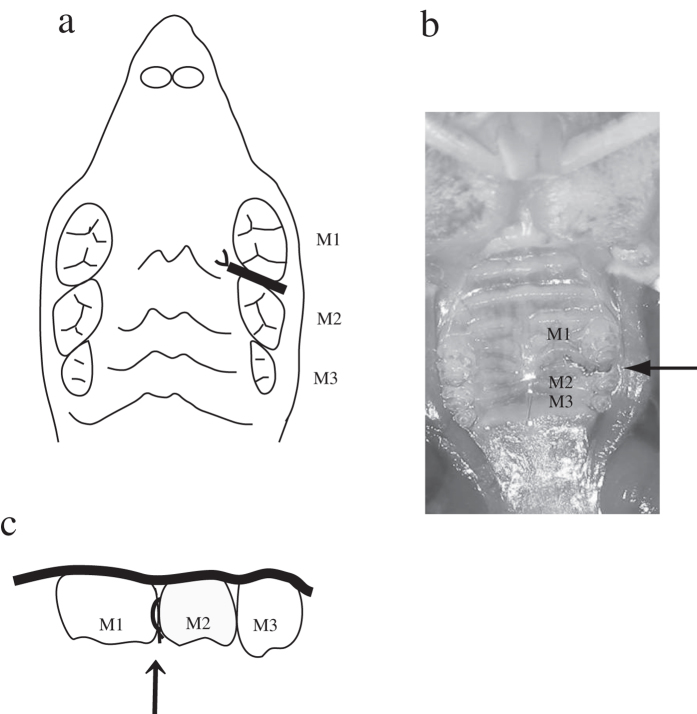
Position of ligature wire placement in mice. (**a**) Position of ligature wire placement, occlusal view. (**b**) Ligature wire was tied around contact point between first molar and second molar. (**c**) Position of ligature wire placement, buccal view. Abbreviations: M1, first molar; M2, second molar; M3, third molar.

Mice were anesthetized via intraperitoneal administration of pentobarbital, and a 0.1-mm-diameter stainless steel ligature wire (Nilaco Corporation, Tokyo, Japan) was placed around the contact point between the left maxillary first molar (M1) and second molar (M2), following the method by Mizuno *et al.* (Fig. 5)^19^. RMA sodium salt (1 mg/kg) was administered intraperitoneally twice daily beginning 3 days before ligature application to 12 animals in the OPG KO mice and 12 WT mice; the remaining 12 OPG KO mice and 12 WT mice did not receive RMA sodium salts. The duration and interval of administration was determined according to a previous study^28^.

### Micro-computed tomography scanning

**Figure 6:**
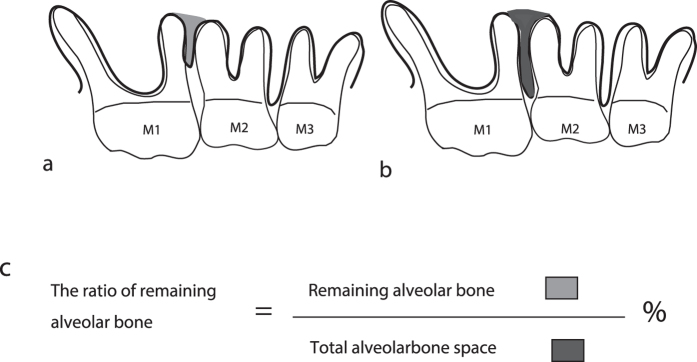
Schematic diagram of the percentage of remaining alveolar bone between M1 and M2 in mice. (**a**) Schematic diagram of the space between the remaining alveolar bone. (**b**) Schematic diagram of the total alveolar bone space. (**c**) Formula for calculating the percentage of remaining alveolar bone. Abbreviations: M1, first molar; M2, second molar; M3, third molar.

At 0 days and at 4 and 8 weeks, maxillae were examined by micro-computed tomography (CT) using a R_mCT system (Rigaku Corporation, Tokyo, Japan). X-ray images of the maxilla were taken at 90 kV, 88 μA, for 2 min, at a picture element size of 20 × 20 × 20 μm. The degree of alveolar bone resorption was analysed according to a modified version of the method reported by Park *et al.*^30^. Briefly, alveolar bone volume between M1 and M2 was measured using TRI/3D-BON software (Ratoc System Engineering Co., Ltd, Tokyo, Japan). Following the method of Mizuno *et al.*^28^, the total alveolar bone space was defined as the space from the cementoenamel junction line to the root apex line. The percentage of the remaining alveolar bone was calculated by dividing its volume by the total alveolar bone volume (Fig. 6).

### Pathological analysis

**Figure 7:**
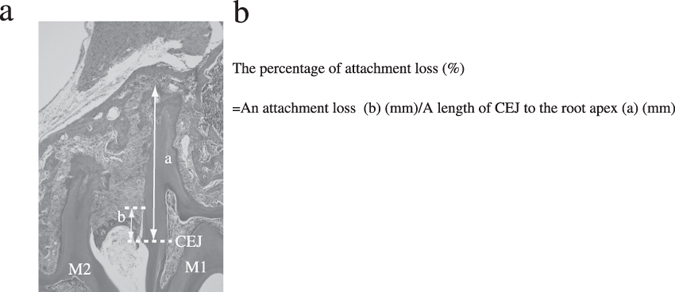
Schematic diagram of the percentage of attachment loss in mice. (**a**) Schematic diagram of the percentage of attachment loss from the cementoenamel junction (CEJ) to the root apex. (**b**) Formula for calculating the percentage of attachment loss Key: (**a**) length of CEJ to root apex; (**b**) area of attachment loss; M1, first molar; M2, second molar; CEJ, cementoenamel junction.

Collected maxillae were fixed in 10% neutral buffered formalin and decalcified in 10% EDTA (pH 7.2) at 4 °C for approximately 4 weeks. Paraffin blocks were prepared according to standard methods and cut into 5-mm-thick mesiodistal serial sections. Tissue observation sites were selected at points where all molar roots could be observed. Next, hematoxylin-eosin (HE) staining and tartrate-resistant acid phosphatase (TRAP) staining with an acid phosphatase leukocyte kit (Sigma, St. Louis, MO, USA) were performed, and stained sections were observed by light microscopy. Osteoclast counts were performed for TRAP-stained sections of the alveolar bone surface at the M1 to M2 interval. TNF-α, IL-1β, and IL-6 immunostaining were also performed. Immunostaining were conducted using the histofine simple stain mouse MAX-PO and histofine simple stain DAB substrate kits (Nichirei Bioscience Inc., Tokyo, Japan) with anti-TNF alpha (ab6671, TNF-α: 1/200, Abcam Inc., Tokyo, Japan), anti-IL-1β (H-153), Human, Rabbit-Poly (SC-7884, IL1-β: 1/150. SCB Santa Cruz Biotechnology, Inc., CA, USA), and anti-IL-6 antibody (ab83339, IL-6: 1/250, Abcam Inc.). Stained sections were observed by light microscopy. Following Rogers *et al.*^29^, the sections were scored as 1, 2, 3, or 4, indicating 0–20%, 20–40%, 40–60%, and >60% positive staining, respectively. Sections were then compared with each ratio of attachment loss. (Fig. 7a, b)

### Statistical analysis

All data are presented as means ± SEM, and statistical analysis was carried out by one-way analysis of variance (ANOVA; Tukey’s multiple comparison test). All statistical analysis were performed with Prism Version 5 (GraphPad Software Inc., San Diego, CA, USA), and P values < 0.05 were considered to be significant.

## Additional Information

**How to cite this article**: Mizuno, M. *et al.* Reveromycin A Administration Prevents Alveolar Bone Loss in Osteoprotegerin Knockout Mice with Periodontal Disease. *Sci. Rep.* **5**, 16510; doi: 10.1038/srep16510 (2015).
